# Sex Differences in Adult ADHD: A Multidimensional Multi-Informant Observational Study

**DOI:** 10.3390/jcm15155954

**Published:** 2026-07-30

**Authors:** Anna Magnesa, Elena Costagli, Francesco De Dominicis, Ugo De Rosa, Anna Iriti, Alessandra Petrucci, Berenice Rimoldi, Pierpaolo Medda, Elisa Schiavi, Giulio Emilio Brancati, Giulio Perugi

**Affiliations:** 1 Department of Clinical and Experimental Medicine, University Hospital of Pisa, 56126 Pisa, Italy; anna.magnesa@gmail.com (A.M.); giulio.perugi@med.unipi.it (G.P.); 2 Mental Health Centre, Local Health Unit 2, Via San Carlo 2, 06049 Spoleto, Italy; 3 Mental Health Centre, Local Health Unit 2, Viale Trieste 68, 05100 Terni, Italy; 4 Psychiatry Unit 2, Azienda Ospedaliero-Universitaria Pisana, Via Roma 67, 56126 Pisa, Italy

**Keywords:** ADHD, sex, emotional dysregulation, comorbidity, assessment

## Abstract

**Background/Objectives**: Sex differences in adult attention-deficit/hyperactivity disorder (ADHD) remain insufficiently characterized, particularly in clinically referred samples assessed through multidimensional and multi-informant approaches. This study investigated sex differences in clinical presentation, psychiatric comorbidity, psychometric features, and ADHD severity in adults with ADHD, with a specific focus on emotional dysregulation and rating-source effects. **Methods**: A total of 212 consecutively recruited adults with ADHD, including 93 females and 119 males, underwent a comprehensive assessment at a tertiary referral service. Sex differences were examined across sociodemographic, clinical, comorbidity, functional, and psychometric domains. ADHD severity was assessed using both self-rated and observer-rated measures. Regression, mediation, and repeated-measures mixed-effects models were fitted to examine the role of emotional dysregulation in the association between sex and ADHD severity. **Results**: Females showed a higher frequency of predominantly inattentive presentation; higher rates of mood, anxiety, and eating disorders; and more pronounced emotional dysregulation, affective temperamental traits, personality-related impairment, and biological rhythm disturbances. Observer-rated ADHD severity did not differ by sex, while females reported higher self-rated ADHD severity. Emotional dysregulation was associated with both observer-rated and self-rated ADHD severity, with a stronger association for self-rated symptoms. Mediation analysis showed a significant indirect effect of female sex on self-reported ADHD severity through emotional dysregulation, accounting for 79.4% of the total association. Mixed-effects models further indicated that sex differences in ADHD severity varied according to rating source and persisted, although attenuated, after accounting for emotional dysregulation. **Conclusions**: Adult females with ADHD showed a different clinical profile, characterized by greater internalizing comorbidity, emotional dysregulation, personality-related impairment, and biological rhythm disturbances. Emotional dysregulation accounted for a substantial part of the higher self-reported ADHD severity observed among females, while rating-source effects suggested that sex differences in adult ADHD may partly depend on how symptom severity is assessed.

## 1. Introduction

Attention-deficit/hyperactivity disorder (ADHD) is a neurodevelopmental disorder characterized by developmentally inappropriate levels of inattention, hyperactivity, and impulsivity. Although most frequently identified during childhood, ADHD often persists into adulthood, where it is associated with substantial clinical heterogeneity, functional impairment, and high rates of psychiatric comorbidity [1,2]. In population-based studies, adult ADHD has an estimated prevalence of approximately 2.5%, although estimates vary according to diagnostic criteria and assessment methods [3].

Sex differences in ADHD have been increasingly recognized. Girls and women may show less overt hyperactive/impulsive manifestations, more prominent inattentive symptoms, and greater affective and internalizing features, potentially contributing to under-recognition and delayed diagnosis [4,5,6]. In adulthood, women receiving a late ADHD diagnosis have described compensatory strategies, diagnostic complexity, and co-occurring emotional difficulties as factors that may have masked ADHD identification, with long-lasting consequences for self-esteem, interpersonal relationships, and psychosocial functioning [7,8].

Beyond differences in core ADHD presentation, sex differences may also involve psychiatric comorbidity. Previous studies suggest that females with ADHD tend to show higher internalizing and affective comorbidity, whereas males may be more frequently characterized by substance use and externalizing disorders [5,6,9,10].

Emotional dysregulation (ED) is particularly relevant in adult ADHD, being highly prevalent and clinically impairing, while only partially overlapping with core ADHD symptoms and psychiatric comorbidity [11,12]. Recent evidence suggests that ADHD with ED may not simply reflect a more severe form of ADHD, but rather a clinically meaningful phenotype characterized by negative emotionality and affective comorbidity [13]. In some studies, females with ADHD have shown higher rates or greater severity of ED than males, both in adult samples and across development [14,15].

Although previous studies have documented sex differences in ADHD presentation, psychiatric comorbidity, and ED, these dimensions have often been examined separately, and relatively little is known about how they converge within a comprehensively characterized adult clinical sample. Moreover, although the clinical assessment of adult ADHD requires the integration of self-report and informant-report information [2,6], it remains insufficiently understood whether sex differences in ADHD severity vary according to rating source and to what extent ED contributes to discrepancies between self-reported and observer-rated symptoms.

To address these gaps, the present study investigated sex differences in a clinical sample of adults with ADHD using a multidimensional and multi-informant assessment that integrated clinical, functional, personality-related, biological-rhythm, self-report, informant-report, and clinician-rated measures. We aimed to characterize sex-related differences across these domains, to examine whether ED statistically accounted for the association between female sex and self-reported ADHD severity, and to determine whether sex differences in ADHD severity varied according to rating source. We hypothesized that females would show a broader internalizing profile, that sex differences would be more evident in self-reported than in observer-rated ADHD severity, and that ED would be associated with self-reported but not observer-rated ADHD severity.

## 2. Materials and Methods

### 2.1. Participants

This observational, cross-sectional study was conducted at the outpatient service for neurodevelopmental disorders of Psychiatry Unit 2, University Hospital of Pisa, between July 2020 and July 2025. The service functions as a tertiary referral center specializing in the diagnostic assessment of adult ADHD. Participants were consecutively recruited from adult patients referred for the diagnostic evaluation of suspected ADHD or for the reassessment of a previously established ADHD diagnosis. Eligibility criteria included age 18 years or older and a confirmed diagnosis of ADHD, as defined by the Diagnostic and Statistical Manual of Mental Disorders, Fifth Edition, Text Revision (DSM-5-TR), at baseline assessment [16].

A history of psychosis, current (hypo)manic or major depressive episodes, and intellectual disability according to DSM-5-TR criteria were considered exclusionary conditions. The final sample comprised 212 adults.

All participants provided written informed consent before enrollment. The study was conducted in accordance with the Declaration of Helsinki and was approved by the Ethics Committee of the University of Pisa (ID: ADHD-MOOD).

### 2.2. Clinical Assessment

At baseline, all participants underwent a comprehensive clinical assessment conducted by psychiatry trainees with specific expertise in neurodevelopmental disorders, under the supervision of senior psychiatrists. ADHD diagnosis was established or confirmed using the Diagnostic Interview for ADHD in Adults (DIVA), a semi-structured clinician-administered interview assessing ADHD symptoms during both childhood and adulthood [17,18]. Both newly diagnosed individuals and patients with a previously established ADHD diagnosis were included; in the latter group, the DIVA was administered to reassess ADHD according to current diagnostic standards and to ensure diagnostic consistency across the sample.

Between July 2020 and January 2024, the DIVA 2.0 was employed [18]. Although this version was originally developed according to DSM-IV criteria, final diagnostic decisions were reformulated in accordance with DSM-5-TR criteria, requiring at least five current symptoms of inattention and/or hyperactivity/impulsivity in adulthood, evidence that several symptoms had been present before 12 years of age, clinically significant impairment, and exclusion of alternative explanatory conditions [16]. From January 2024 onward, the Italian version of the DIVA-5, which directly reflects DSM-5 criteria and is consistent with DSM-5-TR criteria, was adopted [19]. Diagnostic conclusions were based on an integrated clinical formulation combining developmental history, current symptomatology, functional impairment, longitudinal course, and collateral information from relatives or partners, whenever available.

During the clinical interview, sociodemographic information was systematically collected, including sex, age, educational attainment, years of schooling, history of repeated grades, and marital status. A detailed psychiatric family history, with particular attention to first-degree relatives, was also obtained. Current and lifetime psychiatric comorbidities were assessed according to DSM-5-TR criteria and covered the main domains of adult psychopathology, including mood disorders, anxiety disorders, obsessive-compulsive and related disorders, eating disorders, substance use disorders, and personality disorders [16].

Lifetime psychiatric diagnoses were established through a comprehensive clinical evaluation integrating symptom history, longitudinal psychopathological reconstruction, contextual clinical information, family psychiatric history, and previous psychopharmacological treatments. An operationalized diagnostic approach was applied to cyclothymic disorder. Specifically, a lifetime history of manic episodes was considered exclusionary, whereas previous major depressive or hypomanic episodes were not regarded as incompatible with a cyclothymic course when embedded within a longstanding oscillatory affective pattern. Consistent with Akiskal and Mallya’s criteria, onset before 21 years of age was required to exclude iatrogenic or residual mood instability [20].

### 2.3. Psychometric Measures

A battery of standardized self-report, informant-report, and clinician-rated instruments was administered to assess ADHD symptom severity, executive functioning, psychosocial functioning, ED, impulsivity, affective temperament, hypomanic spectrum features, personality pathology, and biological rhythms.

Self-reported ADHD symptom severity was assessed using the Adult ADHD Self-Report Scale version 1.1 (ASRS v1.1), a World Health Organization self-report instrument developed to assess the frequency of current ADHD symptoms in adults [21]. Informant-reported ADHD symptom severity was assessed using the Conners’ Adult ADHD Rating Scales–Observer: Screening Version (CAARS-O:SV), a standardized observer-based measure of adult ADHD symptoms [22]. In the present study, the CAARS-O:SV ADHD symptoms total score was used as the observer-rated index of ADHD severity.

Executive functioning in everyday life was assessed using the informant-report form of the Behavior Rating Inventory of Executive Function–Adult Version (BRIEF-A), an ecologically oriented measure of executive dysfunction in real-world settings [23,24]. The BRIEF-A includes 75 items grouped into nine non-overlapping clinical scales and yields three main composite indices: the Global Executive Composite (GEC), reflecting overall executive dysfunction; the Behavioral Regulation Index (BRI), indexing the ability to regulate behavioral and emotional responses; and the Metacognition Index (MI), capturing higher-order executive processes, including initiation, working memory, planning, organization, and task monitoring.

Psychosocial functioning was clinician-rated using the Functioning Assessment Short Test (FAST), a brief instrument designed to assess impairment across major domains of daily functioning, including autonomy, occupational functioning, cognitive functioning, financial issues, interpersonal relationships, and leisure activities [25,26].

ED was assessed using the Reactivity, Intensity, Polarity, and Stability questionnaire, 40-item version (RIPoSt-40), a self-report instrument developed to measure multiple facets of affective dysregulation [27]. The RIPoSt-40 includes four subscales assessing affective instability, emotional impulsivity, negative emotionality, and positive emotionality. The affective instability, emotional impulsivity, and negative emotionality subscales can also be summed to obtain a second-order Negative Emotion Dysregulation score (RIPoSt-NED), which was used as the main index of ED in the present analyses. Trait impulsivity was assessed with the Barratt Impulsiveness Scale-11 (BIS-11), a widely used self-report measure of impulsivity traditionally organized around attentional, motor, and non-planning components [28].

Affective temperamental traits were measured using the Temperament Evaluation of Memphis, Pisa, Paris, and San Diego–Münster version (TEMPS-M), which assesses depressive, cyclothymic, hyperthymic, irritable, and anxious temperamental dispositions relevant to mood-spectrum psychopathology [29,30]. Lifetime hypomanic spectrum features were screened using the Hypomania Checklist-32 (HCL-32), a self-report instrument developed to detect lifetime manifestations of hypomanic activation and broader bipolar-spectrum phenomenology [31]. In addition to the total score, the Active/Elated and Irritable/Risk-Taking factor scores were analyzed.

Personality pathology was investigated within the framework of the Alternative Model for Personality Disorders retained in Section III of the DSM-5-TR [16]. Severity of personality dysfunction was assessed using the Level of Personality Functioning Scale–Brief Form 2.0 (LPFS-BF 2.0), a 12-item self-report measure providing a dimensional estimate of impairment in self-functioning and interpersonal functioning [32,33]. Maladaptive personality traits were measured with the Personality Inventory for DSM-5 Brief Form Plus (PID5BF+), a brief dimensional instrument aligned with both the DSM-5 maladaptive trait model and the ICD-11 trait framework, assessing negative affectivity, detachment, antagonism, disinhibition, anankastia, and psychoticism [34,35].

Finally, disturbances in circadian and daily biological rhythms were evaluated using the Biological Rhythms Interview of Assessment in Neuropsychiatry (BRIAN), which explores dysregulation across clinically relevant domains such as sleep, daily activity, social rhythms, and eating-related rhythmicity [36].

### 2.4. Statistical Analysis

All statistical analyses were performed using R Statistical Software Version 4.3.1 (R Foundation for Statistical Computing, Vienna, Austria). Descriptive statistics were used to summarize the sociodemographic, clinical, comorbidity, and psychometric characteristics of the sample, both in the overall sample and after stratification by sex. When scoring questionnaires and rating scales, missing items from subscales with less than 15% missing data were imputed using the respondent’s median of the completed items within the same subscale. This procedure was limited to isolated missing items within otherwise completed questionnaires; whole-scale scores were not imputed, and analyses involving unavailable scores were conducted using available cases. Overall-sample descriptive statistics and missing-data proportions are provided in Supplementary Tables S1–S4. Comparisons between participants with and without available scores are reported in the Supplementary Materials. Informant type for the CAARS-O:SV and BRIEF-A was categorized as parent, partner, or other relative, and its distribution was compared between female and male participants using Pearson’s chi-squared test.

The distribution of continuous variables was examined using the Shapiro–Wilk test. Because the variables entered into the comparative analyses did not satisfy normality assumptions, all continuous and ordinal variables were treated as non-normally distributed. Bivariate sex comparisons were performed using the Wilcoxon rank-sum test for continuous and ordinal variables, and Pearson’s chi-squared test or Fisher’s exact test for categorical variables. Standardized mean differences (SMDs) were computed as effect-size estimates for between-group contrasts.

Post hoc analyses were performed for multinomial categorical variables showing a statistically significant omnibus test, namely the ADHD presentation specifier and lifetime mood-disorder profile. Pairwise comparisons across categories were conducted using Fisher’s exact test, adjusted for multiple testing using the Benjamini–Hochberg false discovery rate (FDR) correction.

To account for multiple testing across the 94 bivariate sex comparisons, the Benjamini–Hochberg false discovery rate (FDR) procedure was applied. Raw *p*-values and Benjamini–Hochberg FDR-adjusted *p*-values (*p*_FDR_) were reported.

Ordinary least squares linear regression models were fitted to further examine whether the association between sex and ADHD symptom severity persisted after adjustment for treatment status or ED as measured by RIPoSt-NED. Two outcomes were considered separately: the CAARS-O:SV ADHD symptoms total score and the ASRS v1.1 total score. For each outcome, one model included sex and current treatment status as predictors, whereas a second model included sex and RIPoSt-NED. Models were fitted on model-specific complete cases, and unstandardized regression coefficients with 95% confidence intervals (CIs) and *p*-values were estimated.

A mediation analysis was then conducted to examine whether ED statistically accounted for the association between female sex and self-reported ADHD severity. The model specified female sex as the independent variable, the RIPoSt-NED score as the mediator, and the ASRS v1.1 total score as the outcome. Both the mediator and outcome models were estimated using OLS regression. The average causal mediation effect (ACME), direct effect, total effect, and proportion mediated were obtained using nonparametric bootstrap procedures based on 5000 resamples and percentile 95% CIs. A sensitivity analysis was performed by fitting analogous mediator, outcome, and total-effect models additionally adjusted for age, ADHD presentation specifier, current treatment status, and any lifetime mood disorder. ADHD presentation was entered as predominantly inattentive versus other to ensure stable estimation across bootstrap resamples.

Finally, repeated-measures linear mixed models were used to jointly analyze observer-rated and self-reported ADHD severity. All models included a random intercept for participant and were estimated by restricted maximum likelihood (REML), with degrees of freedom and *p*-values for fixed effects obtained using Satterthwaite’s approximation. For each participant, the CAARS-O:SV ADHD symptoms total score and the ASRS v1.1 total score were standardized within the complete-case sample as z-scores. Rating scale was entered as a within-participant factor, with ASRS v1.1 as the reference scale. Model A regressed the standardized severity z-score on scale, sex, and their interaction, with female sex as the reference category. Model B further included standardized RIPoSt-NED and its interactions, modeling the standardized severity z-score as a function of the three-way scale-by-sex-by-RIPoSt-NED interaction and all corresponding lower-order terms. Models A and B were fitted on the same complete-case sample, so that estimates from the two models were directly comparable. For all fixed effects, coefficients with 95% CIs and Satterthwaite *p*-values were reported.

All statistical tests were two-sided, and the threshold for statistical significance was set at *p* < 0.05.

## 3. Results

### 3.1. Sociodemographic and Clinical Characteristics

The sample comprised 212 adults with ADHD, including 93 females and 119 males, with a median age of 24 years (Tables S1–S4). Females and males did not significantly differ in age, years of education, number of repeated grades, or marital status. Age at first referral was numerically higher in females than in males, although this difference did not reach statistical significance (Table 1).

ADHD presentation differed significantly by sex. Post hoc comparisons indicated that this difference was primarily driven by the contrast between predominantly inattentive and combined presentations (Fisher’s *p*_adj_ < 0.001). Predominantly inattentive presentation was more frequently observed among females than males, whereas combined presentation was more frequent among males than females. Current treatment status showed a non-significant trend toward higher treatment rates among males. No significant sex differences emerged in first-degree family history.

### 3.2. Psychiatric Comorbidities

Significant sex differences were observed in lifetime psychiatric comorbidities (Table 2). Females showed a higher prevalence of any mood disorder than males, while age at onset of mood disorders did not differ between groups. The distribution of lifetime mood disorder diagnoses also differed by sex. Post hoc comparisons showed that the sex distribution for cyclothymic disorder differed significantly from both no mood disorder (Fisher’s *p*_adj_ < 0.001) and bipolar II disorder (Fisher’s *p*_adj_ = 0.018), whereas the contrast with major depressive disorder did not survive correction for multiple testing (Fisher’s *p*_adj_ = 0.059).

Females also showed higher rates of any anxiety disorder, particularly panic disorder. Social anxiety disorder was also more frequent among females at the unadjusted level, but this difference did not remain significant after FDR correction.

Body dysmorphic disorder was more common among females, whereas no significant sex differences emerged for obsessive-compulsive disorder or for the overall obsessive-compulsive and related disorders category.

Eating disorders were significantly more frequent among females, including anorexia nervosa, bulimia nervosa, and binge-eating disorder. Conversely, substance use disorders and impulse-control/conduct disorders were numerically more frequent among males, although these differences did not reach statistical significance.

No significant sex difference emerged for somatic symptom disorders.

### 3.3. Observer- and Clinician-Rated Measures of ADHD Symptom Severity, Executive Dysfunction, and Psychosocial Functioning

No significant sex differences were found in observer-rated ADHD symptom severity on the CAARS-O:SV, including inattention, hyperactivity/impulsivity, ADHD symptoms total score, and ADHD Index (Table 3). Among the 174 participants with available CAARS-O:SV scores, informants were predominantly parents (78.2%), followed by partners (13.2%) and other relatives (8.6%). Informant composition did not differ significantly by participant sex, χ^2^(2) = 4.29, *p* = 0.117, Cramér’s V = 0.16.

With respect to executive dysfunction, no significant sex differences emerged in BRIEF-A composite scores. Among BRIEF-A subscales, however, females showed significantly greater impairment in Emotional Control, whereas no differences were observed in the remaining subdomains. Among the 173 participants with available BRIEF-A scores, informants were predominantly parents (78.6%), followed by partners (13.3%) and other relatives (8.1%), with no significant difference in informant composition by sex, χ^2^(2) = 4.51, *p* = 0.105, Cramér’s V = 0.16.

Clinician-rated psychosocial functioning was comparable in females and males on the FAST total score. Nevertheless, females showed greater FAST Cognitive Functioning impairment, whereas the Leisure Time difference did not survive after FDR correction; no differences emerged in the other FAST domains.

### 3.4. Self-Reported Clinical and Psychometric Features

Several sex differences were observed across self-reported measures (Table 4). Females reported significantly higher ADHD symptom severity on the ASRS v1.1 total score. No significant sex differences were found in BIS-11 impulsivity scores.

On the RIPoSt-40, females scored higher than males Affective Instability, Emotional Impulsivity, Negative Emotionality, and RIPoSt-NED. A significant difference was observed for Positive Emotionality at the unadjusted level. Regarding hypomanic features, females showed higher HCL-32 Active/Elated score. A significant difference was also observed for the HCL-32 total score at the unadjusted level.

Affective temperamental traits also differed by sex. Females showed higher depressive, cyclothymic, and anxious temperamental scores, while males showed higher hyperthymic temperament scores.

With respect to personality measures, females showed greater impairment in LPFS-BF 2.0 Self-Functioning, while the difference in Interpersonal Functioning was significant only at the unadjusted level. On the PID5BF+, females scored higher on Negative Affectivity, whereas males scored higher on Antagonism. At the unadjusted level, females also scored significantly higher in Disinhibition.

Finally, females showed higher BRIAN scores across Sleep, Activity, and Eating rhythm domains, as well as in the total score. A significant difference was also found for the Social rhythm domain at the unadjusted level.

### 3.5. Linear Regression Analyses

Linear regression models were fitted to explore the association of sex, treatment status or ED with ADHD severity (Table S5). In models adjusted for treatment status, male sex was not significantly associated with observer-rated ADHD severity on the CAARS-O:SV, whereas it was associated with lower self-reported ADHD severity on the ASRS v1.1. Being on treatment was significantly associated with lower ASRS v1.1 scores, but not with CAARS-O:SV scores.

In models adjusted for RIPoSt-NED, higher ED severity was significantly associated with both observer-rated and self-reported ADHD severity, with larger coefficients for the ASRS v1.1 than for the CAARS-O:SV. In these models, the association between sex and ASRS v1.1 was no longer significant, whereas male sex was associated with higher CAARS-O:SV scores.

### 3.6. Mediation Analysis

**Figure 1:**
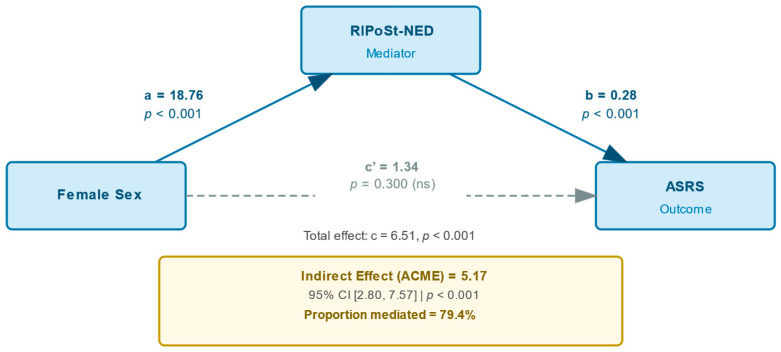
Path diagram of the mediation model testing the indirect association between female sex and self-reported ADHD severity through RIPoSt-NED. Notes: *N* = 209. Female sex was coded as 1 = female and 0 = male. Path coefficients are unstandardized regression coefficients. Solid-line arrows represent the statistically significant paths a and b of the mediation model, whereas the dashed-line arrow represents the non-significant direct effect (c′) after accounting for the mediator. Abbreviations: ACME = average causal mediation effect; ADHD = attention-deficit/hyperactivity disorder; ASRS = Adult ADHD Self-Report Scale v1.1; CI = confidence interval; RIPoSt-NED = Negative Emotion Dysregulation score of the Reactivity, Intensity, Polarity, and Stability Questionnaire–40-item version.

A mediation analysis was performed to examine whether RIPoSt-NED accounted for the association between female sex and self-reported ADHD severity on the ASRS v1.1 (Figure 1 and Table S6). Female sex was significantly associated with higher RIPoSt-NED scores, and RIPoSt-NED was significantly associated with higher ASRS v1.1 scores after adjustment for sex. The total association between female sex and ASRS v1.1 was significant, whereas the direct association was no longer significant after inclusion of RIPoSt-NED in the model.

The indirect effect through RIPoSt-NED was significant (ACME = 5.17, 95% CI = 2.80–7.57, *p* < 0.001), accounting for 79.4% of the total association. Sensitivity analysis additionally adjusted for age, ADHD presentation, treatment status, and any lifetime mood disorder yielded comparable findings, with a significant indirect effect and a similar proportion mediated. Given the cross-sectional design, the estimated indirect effect should be interpreted as statistical mediation and does not establish a causal pathway or the temporal ordering between ED and self-reported ADHD severity.

### 3.7. Self- and Observer-Rated ADHD Severity in Mixed-Effects Models

Repeated-measures mixed-effects models were fitted to analyze self-rated and observer-rated ADHD severity concurrently (Table 5). In Model A, a significant Scale × Sex interaction emerged, indicating that sex differences in ADHD severity varied according to rating source. Specifically, male sex was associated with lower standardized ADHD severity on the self-rated ASRS v1.1, whereas this pattern was not observed for the observer-rated CAARS-O:SV.

In Model B, ED as measured by RIPoSt-NED was significantly associated with higher ADHD severity. After inclusion of ED, the main effect of sex was no longer significant, while the Scale × Sex interaction remained significant, although attenuated. The Scale × ED and Sex × ED interactions were not significant. The three-way Scale × Sex × ED interaction showed only a non-significant trend.

## 4. Discussion

In this study, adult females with ADHD showed a different clinical and psychometric profile compared to males with ADHD. This profile was characterized by a higher prevalence of predominantly inattentive presentation, and mood, anxiety, and eating disorders; higher levels of ED and related affective temperamental traits; personality-related impairment; and biological rhythm disturbances. Overall, effect sizes were generally moderate, with some larger effects, indicating group-level tendencies rather than separable sex-specific phenotypes. Observer-rated ADHD severity did not differ by sex, despite higher self-rated ADHD symptom severity among females. While the magnitude of sex differences in ADHD severity appeared to depend, at least in part, on the source of assessment, regression, mediation, and mixed-effects models suggested that higher levels of ED accounted for a substantial part of the association between female sex and self-reported ADHD severity.

The higher frequency of predominantly inattentive presentation observed among females in the present study is consistent with previous reports suggesting that ADHD in girls and women may be less externally disruptive and, consequently, less readily identified [5,6]. Males with ADHD are more likely to display hyperactive and impulsive behaviors that may facilitate referral and diagnosis, whereas the identification of ADHD in females may be complicated by less overt symptom expression and a greater internalizing burden [4,6]. This interpretation is further supported by qualitative evidence from women diagnosed with ADHD in adulthood, who frequently described delayed recognition of their symptoms, diagnostic complexity, compensatory strategies, and co-occurring emotional symptoms as factors that masked ADHD identification [8].

Sex differences in psychiatric comorbidity further supported the presence of a more internalizing clinical profile among females. The higher prevalence of mood disorders, particularly cyclothymic disorder, together with anxiety and eating disorders, suggests that ADHD symptoms in females may be embedded within a broader mood-spectrum and ED phenotype. This interpretation is consistent with previous evidence showing higher rates of internalizing problems, anxiety, depressive symptoms, and affective comorbidity among females with ADHD [5,9,10]. Conversely, substance use disorders and impulse-control/conduct disorders were numerically more frequent among males, although these differences did not reach statistical significance, partially mirroring previous evidence of higher substance use and externalizing comorbidity among males with ADHD [5,9,10]. The observed sex differences in participants’ lifetime psychiatric comorbidity were not paralleled by significant differences in reported first-degree family history across the assessed diagnostic domains.

A clinically relevant finding was the discrepancy between self-rated and observer-rated ADHD severity. Females reported higher ADHD symptom severity on the ASRS v1.1, whereas no sex differences emerged on the observer-rated CAARS-O:SV. Informant-type distributions for the observer-rated measures did not differ significantly by sex, suggesting that differential informant composition was unlikely to explain the observed rating-source discrepancy. Recent evidence indicates that sex differences in core ADHD symptom severity are generally small and may vary according to sample characteristics and assessment method [37,38]. Previous studies in adults with ADHD have also found only small-to-moderate concordance between self- and informant ratings, suggesting that these sources capture partly different aspects and contexts of symptom expression rather than one perspective being consistently more accurate [39].

Other factors may also be involved in the discrepancy between severity ratings. For instance, current treatment status was associated with lower self-reported, but not observer-rated, ADHD severity. However, because treatment was not randomized and was assessed cross-sectionally, this association cannot be interpreted as a pharmacological treatment effect. Adjustment for treatment status did not account for the observed sex difference in self-reported ADHD severity, and the adjusted mediation analysis yielded comparable findings. An alternative contribution may lie in the different composition of the scales. Since the ASRS v1.1 and CAARS-O:SV are not parallel forms of the same instrument, their divergence cannot be attributed exclusively to rating source.

ED appeared to be a central dimension of the female ADHD profile observed in the present study. Females scored higher across ED dimensions and showed greater observer-rated impairment in Emotional Control, suggesting that this pattern was not confined to self-perceived emotional difficulties. They also showed higher depressive, cyclothymic, and anxious temperamental scores, whereas males showed higher hyperthymic traits. Taken together, these findings highlight a tendency toward more pronounced affective instability among females, consistent with previous reports of greater ED among adult women with ADHD and its persistence across development in girls with ADHD [14,15], as well as with evidence linking cyclothymic temperament and ED in adults with ADHD [40].

Secondary analyses further clarified the role of ED in the rating-source discrepancy. Previous work has separately examined ED in relation to ADHD symptoms [11,12,13], sex differences in ED [14,15], and concordance between self- and informant ratings [39], whereas the joint role of sex and ED across rating sources had remained largely unexplored until now. In our sample, higher ED was associated with greater ADHD severity across both self- and observer-rated measures. After adjustment for ED, the higher self-reported ADHD severity observed in females was no longer significant, whereas males showed higher observer-rated severity, suggesting that ED primarily accounted for the female elevation in self-reported severity. Mediation analyses indicated that ED statistically accounted for a substantial part of the association between female sex and higher self-reported ADHD severity. In the mixed-effects model, inclusion of ED attenuated the Scale × Sex interaction, although the interaction remained significant, indicating that ED did not fully account for the sex-related rating-source discrepancy.

Sex differences also emerged in personality functioning and maladaptive personality traits. Females scored higher on Negative Affectivity and males on Antagonism, a pattern also reported in general population samples assessed with the PID-5 brief form, in which men additionally scored higher on Disinhibition [41]. Females also showed greater impairment in self-functioning, consistent with recent evidence that personality-functioning difficulties contribute to functional impairment in women with ADHD beyond the effects of co-occurring depression, anxiety, and ADHD symptoms [42].

Finally, females showed greater disturbances in sleep, activity, and eating rhythms, as well as in overall biological rhythm functioning. Sleep and circadian rhythm problems are common in adult ADHD and have been associated with psychiatric comorbidity [43,44], while previous work specifically linked sleep–wake rhythm disturbances with cyclothymic temperament and ED in adults with ADHD and/or cyclothymia [40]. Beyond these dimensions, the impact of female-specific hormonal fluctuations, such as menstrual-cycle phase, pregnancy/postpartum status, perimenopause, menopause, and hormonal treatments, warrant clinical attention, given emerging evidence that hormonal fluctuations may influence ADHD symptoms, emotional symptoms, and perceived medication response in females with ADHD [45].

Strengths of the present study include the relatively large clinical sample of adults with ADHD, consecutively recruited in a tertiary referral setting and systematically assessed through a comprehensive clinical evaluation including semi-structured ADHD assessment. In addition, the study provided a broad multidimensional characterization of clinical, comorbidity, functional, and psychometric domains, using self-report, informant-report, and clinician-rated instruments. The availability of both self-rated and observer-rated ADHD severity measures, together with regression, mediation, and mixed-effects models, allowed the direct examination of rating-source effects and a more detailed assessment of the role of ED in sex differences in ADHD severity.

Some limitations should be acknowledged. First, the cross-sectional design precludes conclusions regarding the temporal or causal relationships among sex, ED, and ADHD severity. Accordingly, mediation findings should be interpreted as evidence of statistical mediation rather than causal mechanisms. Second, the study was conducted at a single tertiary referral service. Patients referred to highly specialized ADHD services are likely to present greater diagnostic complexity and psychiatric comorbidity than adults with ADHD identified in community, primary-care, or less specialized clinical settings. The clinical profiles, comorbidity burden, and psychometric characteristics observed in the present sample may therefore partly reflect referral-related selection and should not be assumed to generalize to the broader population of adults with ADHD. Third, missing data represent an additional limitation. Missingness in CAARS-O:SV and BRIEF-A scores was not associated with sex, age, self-reported ADHD severity, or ED, although residual selection related to unmeasured factors cannot be excluded. Missingness was higher and age-related for FAST Occupational Functioning and the FAST total score; findings involving these measures should therefore be interpreted cautiously. Importantly, BRIEF-A and FAST were not included in the principal multivariable models, and the mixed-effects models were fitted on the same complete-case sample. Fourth, the large number of exploratory bivariate comparisons may have increased the risk of type I error. Although most significant findings survived correction for multiple comparisons, findings that did not remain significant after correction should be considered exploratory and require independent replication. Fifth, several psychometric domains were assessed using self-report instruments. In particular, both the mediator, RIPoSt-NED, and the primary outcome, ASRS v1.1 total score, in the mediation analysis were self-reported; shared-method variance may therefore have contributed to the estimated indirect association. Finally, hormone-related variables were not collected, precluding examination of their potential contribution to the observed sex differences.

## 5. Conclusions

In conclusion, the present study suggests that sex differences in adult ADHD extend beyond core inattentive and hyperactive/impulsive symptom dimensions. Compared with males, females with ADHD showed a different clinical and psychometric profile characterized by a higher frequency of predominantly inattentive presentation, greater internalizing and mood-spectrum comorbidity, higher ED, personality-related impairment, and biological rhythm disturbances. At the same time, observer-rated ADHD severity did not differ by sex. ED partially explained the association between female sex and higher self-reported severity, while sex differences across self- and observer-rated measures remained significant. Overall, these findings support the view that adult ADHD in females may be embedded within a broader affective and internalizing psychopathological context, in which ED and affective comorbidity contribute to subjective symptom severity and clinical presentation. Longitudinal studies are warranted to clarify the directionality of these associations and their implications for diagnostic characterization and treatment planning.

## Figures and Tables

**Table 1:** Sociodemographic, clinical, and first-degree family history characteristics of the sample according to sex.

	Females (*n* = 93)	Males (*n* = 119)				
Variables	Median [IQR]/*n* (%)	Median [IQR]/*n* (%)	U/χ^2^	SMD	*p*	*p*_FDR_
**Sociodemographic characteristics**						
Age (years)	24.00 [21.00, 30.00]	23.00 [20.00, 29.00]	4975.50	0.215	0.207	0.364
Education (years)	13.00 [13.00, 14.00]	13.00 [13.00, 15.00]	5390.50	0.106	0.903	0.944
Repeated grades	0.00 [0.00, 1.00]	0.00 [0.00, 1.00]	5338.00	0.053	0.933	0.955
**Marital status**			–	0.204	0.309	0.480
Single	82 (88.2)	111 (93.3)				
Married	8 (8.6)	7 (5.9)				
Divorced	3 (3.2)	1 (0.8)				
**Clinical characteristics**						
Age at first referral (years)	18.00 [14.00, 22.00]	15.00 [8.00, 21.00]	4289.00	0.279	0.063	0.145
**ADHD Presentation**			–	0.545	**<0.001**	**0.005**
Combined	32 (34.4)	71 (60.2)				
Predominantly inattentive	59 (63.4)	44 (37.3)				
Predominantly hyperactive/impulsive	2 (2.2)	3 (2.5)				
Treatment status (on treatment)	11 (13.3)	28 (25.5)	3.64	0.313	0.056	0.143
**Family history (first degree)**						
Mood disorders	54 (58.1)	63 (52.9)	0.37	0.103	0.545	0.657
Anxiety disorders	24 (25.8)	30 (25.2)	0.00	0.014	1.000	1.000
Neurodevelopmental disorders	21 (22.6)	18 (15.1)	1.47	0.191	0.226	0.380
Substance use disorders	7 (7.5)	17 (14.3)	1.75	0.218	0.186	0.343
Suicide attempt	8 (8.6)	4 (3.4)	1.79	0.222	0.181	0.339
Psychotic disorders	4 (4.3)	2 (1.7)	–	0.154	0.408	0.564

Notes: *p* and *p*_FDR_ values < 0.05 are shown in bold. Abbreviations: ADHD = attention-deficit/hyperactivity disorder; IQR = interquartile range; SMD = standardized mean difference; *p*_FDR_ = *p*-value adjusted using the Benjamini–Hochberg false discovery rate procedure.

**Table 2:** Lifetime psychiatric comorbidities according to sex.

	Females (*n* = 93)	Males (*n* = 119)				
Variables	Median [IQR]/*n* (%)	Median [IQR]/*n* (%)	U/χ^2^	SMD	*p*	*p*_FDR_
**Any mood disorder**	78 (83.9)	73 (61.3)	11.85	0.522	**0.001**	**0.004**
Mood disorder			–	0.745	**<0.001**	**<0.001**
None	15 (16.1)	46 (38.7)				
Bipolar I disorder	2 (2.2)	3 (2.5)				
Bipolar II disorder	18 (19.4)	30 (25.2)				
Cyclothymic disorder	54 (58.1)	30 (25.2)				
Major depressive disorder	4 (4.3)	10 (8.4)				
Age at onset (years)	16.00 [13.50, 19.50]	16.00 [14.00, 20.00]	2238.50	0.005	0.458	0.593
**Any anxiety disorder**	62 (66.7)	50 (42.0)	11.76	0.511	**0.001**	**0.004**
Separation anxiety disorder	10 (10.8)	11 (9.2)	0.02	0.050	0.894	0.944
Panic disorder	35 (37.6)	21 (17.6)	9.73	0.459	**0.002**	**0.009**
Agoraphobia	8 (8.6)	3 (2.5)	–	0.268	0.062	0.145
Social anxiety disorder	26 (28.0)	18 (15.1)	4.47	0.316	**0.034**	0.095
Generalized anxiety disorder	23 (24.7)	18 (15.1)	2.50	0.242	0.114	0.249
**Any obsessive-compulsive or related disorder**	10 (10.8)	6 (5.0)	1.69	0.213	0.194	0.350
Obsessive-compulsive disorder	2 (2.2)	5 (4.2)	–	0.117	0.470	0.597
Body dysmorphic disorder	9 (9.7)	1 (0.8)	–	0.404	**0.006**	**0.023**
**Any eating disorder**	25 (26.9)	7 (5.9)	16.36	0.592	**<0.001**	**<0.001**
Anorexia nervosa	6 (6.5)	0 (0.0)	–	0.371	**0.006**	**0.023**
Bulimia nervosa	6 (6.5)	0 (0.0)	–	0.371	**0.006**	**0.023**
Binge eating disorder	18 (19.4)	7 (5.9)	7.86	0.414	**0.005**	**0.022**
**Any substance use disorder**	19 (20.4)	39 (32.8)	3.40	0.282	0.065	0.145
Alcohol use disorder	9 (9.7)	16 (13.4)	0.40	0.118	0.529	0.646
Cannabis use disorder	16 (17.2)	29 (24.4)	1.20	0.177	0.273	0.427
Cocaine use disorder	6 (6.5)	15 (12.6)	1.58	0.211	0.209	0.364
Opioid use disorder	2 (2.2)	2 (1.7)	–	0.034	1.000	1.000
Sedative, hypnotic, or anxiolytic use disorder	3 (3.2)	2 (1.7)	–	0.100	0.656	0.746
Stimulant use disorder	3 (3.2)	6 (5.0)	–	0.091	0.734	0.812
**Any impulse-control/conduct disorder**	4 (4.3)	15 (12.6)	3.45	0.302	0.063	0.145
Oppositional defiant disorder	4 (4.3)	15 (12.6)	3.45	0.302	0.063	0.145
Conduct disorder	0 (0.0)	4 (3.4)	–	0.264	0.133	0.277
**Any somatic symptom disorder**	5 (5.4)	3 (2.5)	–	0.147	0.303	0.459

Notes: *p* and *p*_FDR_ values < 0.05 are shown in bold. Abbreviations: IQR = interquartile range; *p*_FDR_ = *p*-value adjusted using the Benjamini–Hochberg false discovery rate procedure; SMD = standardized mean difference.

**Table 3:** Observer- and clinician-rated measures of ADHD symptom severity, executive dysfunction, and psychosocial functioning according to sex.

	Females (*n* = 93)	Males (*n* = 119)				
Variables	Median [IQR]	Median [IQR]	U	SMD	*p*	*p*_FDR_
**CAARS-O:SV**						
Inattention	16.00 [13.00, 21.00]	17.00 [13.00, 22.00]	3535.50	0.081	0.645	0.746
Hyperactivity/Impulsivity	9.00 [5.00, 15.00]	11.00 [6.00, 15.00]	3330.00	0.163	0.276	0.427
ADHD symptoms total	26.00 [19.00, 34.00]	27.00 [21.00, 36.00]	3423.50	0.142	0.422	0.576
ADHD Index	21.00 [16.00, 26.00]	21.00 [15.00, 26.00]	3563.00	0.085	0.706	0.791
**BRIEF-A (Informant)**						
Inhibit	14.00 [11.00, 17.00]	15.00 [12.00, 18.00]	3257.50	0.193	0.226	0.380
Shift	13.00 [11.00, 14.00]	12.00 [10.00, 15.00]	3271.50	0.234	0.242	0.394
Emotional Control	21.00 [18.00, 25.00]	18.00 [14.00, 23.00]	2694.50	0.448	**0.003**	**0.015**
Self-Monitor	10.00 [8.00, 13.00]	11.00 [8.00, 14.00]	3444.00	0.100	0.525	0.646
Initiate	18.00 [16.00, 20.00]	17.00 [14.00, 20.00]	3185.50	0.252	0.152	0.298
Working Memory	17.00 [14.00, 20.00]	16.00 [14.00, 19.00]	3378.00	0.148	0.402	0.564
Plan/Organize	21.00 [19.00, 24.00]	21.00 [19.00, 24.25]	3614.50	0.010	0.913	0.944
Task Monitor	13.00 [11.00, 14.00]	13.00 [12.00, 15.00]	3356.50	0.115	0.363	0.535
Organization of Materials	19.00 [16.00, 22.00]	19.00 [16.00, 22.00]	3401.50	0.161	0.443	0.588
Behavioral Regulation Index (BRI)	57.00 [53.00, 66.00]	57.00 [48.00, 67.25]	3283.50	0.166	0.260	0.415
Metacognition Index (MI)	89.00 [80.00, 98.00]	89.00 [77.25, 98.25]	3527.50	0.116	0.708	0.930
Global Executive Composite (GEC)	146.00 [134.00, 159.00]	144.50 [122.50, 163.00]	3422.50	0.151	0.485	0.686
**FAST (Clinician)**						
Autonomy	5.00 [4.00, 7.00]	5.00 [3.00, 7.00]	3618.50	0.030	0.774	0.847
Occupational Functioning	8.00 [6.00, 11.00]	7.00 [4.00, 10.00]	1759.50	0.240	0.175	0.337
Cognitive Functioning	9.00 [7.00, 10.00]	7.00 [5.00, 10.00]	3024.00	0.396	**0.011**	**0.035**
Financial Issues	3.00 [1.00, 4.00]	2.00 [1.00, 4.00]	3797.00	0.026	0.901	0.944
Interpersonal Relationships	8.00 [4.00, 10.00]	8.00 [4.50, 11.00]	3547.00	0.099	0.429	0.577
Leisure Time	3.00 [1.00, 4.00]	2.00 [0.00, 3.00]	3145.00	0.291	**0.026**	0.080
Total Score	33.00 [28.00, 42.50]	32.00 [22.50, 43.75]	1764.50	0.121	0.527	0.646

Notes: *p* and *p*_FDR_ values < 0.05 are shown in bold. Abbreviations: ADHD = attention-deficit/hyperactivity disorder; BRI = Behavioral Regulation Index; BRIEF-A = Behavior Rating Inventory of Executive Function–Adult Version; CAARS-O:SV = Conners’ Adult ADHD Rating Scales–Observer: Screening Version; FAST = Functioning Assessment Short Test; GEC = Global Executive Composite; IQR = interquartile range; MI = Metacognition Index; *p*_FDR_ = *p*-value adjusted using the Benjamini–Hochberg false discovery rate procedure; SMD = standardized mean difference.

**Table 4:** Self-reported measures of ADHD symptom severity, impulsivity, emotional dysregulation, affective temperament, personality functioning and traits, and biological rhythm disturbances according to sex.

	Females (*n* = 93)	Males (*n* = 119)				
Variables	Median [IQR]	Median [IQR]	U	SMD	*p*	*p*_FDR_
**ASRS v1.1 Total**	49.00 [42.00, 56.00]	43.00 [32.00, 51.50]	3905.00	0.551	**<0.001**	**0.002**
**BIS-11**						
Attentional	22.00 [19.00, 24.00]	21.00 [19.00, 23.00]	4705.00	0.169	0.149	0.298
Motor	26.00 [23.00, 28.00]	26.00 [23.00, 28.00]	5076.50	0.096	0.658	0.746
Non-Planning	29.00 [26.00, 30.00]	28.00 [27.00, 30.00]	4912.00	0.149	0.621	0.731
Total Score	75.00 [72.00, 78.00]	74.00 [71.00, 77.00]	4768.50	0.029	0.403	0.564
**RIPoSt-40**						
Affective Instability	51.00 [43.00, 61.00]	42.00 [31.25, 57.00]	3781.00	0.557	**<0.001**	**0.002**
Emotional Impulsivity	32.50 [25.00, 38.00]	27.00 [19.00, 34.50]	4082.00	0.455	**0.002**	**0.008**
Negative Emotionality	45.00 [39.00, 52.00]	39.00 [29.50, 47.00]	3675.50	0.648	**<0.001**	**<0.001**
Positive Emotionality	41.50 [33.75, 49.00]	39.00 [28.00, 46.00]	4560.50	0.321	**0.038**	0.102
Negative Emotion Dysregulation	131.00 [107.50, 147.00]	108.50 [82.25, 137.00]	3625.00	0.630	**<0.001**	**<0.001**
**HCL-32**						
Factor 1 (Active/Elated)	13.00 [10.00, 16.00]	11.00 [8.25, 14.00]	4177.00	0.367	**0.006**	**0.023**
Factor 2 (Irritable/Risk-Taking)	5.00 [4.00, 7.00]	5.00 [3.75, 7.00]	4901.50	0.121	0.375	0.543
Total Score	19.00 [14.00, 22.00]	16.00 [13.00, 20.00]	4353.50	0.275	**0.030**	0.087
**TEMPS-M**						
Depressive	23.50 [21.00, 28.25]	23.00 [16.00, 26.00]	4051.50	0.506	**0.002**	**0.008**
Cyclothymic	26.00 [21.75, 31.00]	20.50 [16.00, 27.00]	3689.00	0.594	**<0.001**	**<0.001**
Hyperthymic	19.00 [15.00, 22.00]	21.00 [17.00, 25.00]	4252.50	0.395	**0.007**	**0.025**
Irritable	20.00 [13.00, 24.12]	17.00 [12.00, 22.00]	4786.00	0.195	0.141	0.289
Anxious	24.00 [19.00, 28.25]	16.00 [12.00, 22.00]	2666.50	0.976	**<0.001**	**<0.001**
**LPFS-BF 2.0**						
Self-Functioning	18.00 [14.00, 21.00]	15.00 [12.25, 18.00]	3876.00	0.548	**<0.001**	**0.003**
Interpersonal Functioning	16.00 [13.00, 19.00]	14.00 [11.00, 17.00]	4407.50	0.314	**0.026**	0.080
**PID5BF+**						
Negative Affectivity	3.67 [2.67, 4.17]	2.67 [1.92, 4.00]	3585.00	0.593	**<0.001**	**<0.001**
Detachment	2.00 [1.33, 3.00]	2.00 [1.33, 3.00]	5062.50	0.142	0.460	0.593
Antagonism	1.33 [0.67, 2.33]	2.00 [1.00, 3.00]	4039.50	0.337	**0.011**	**0.035**
Disinhibition	3.33 [2.33, 4.00]	2.83 [2.00, 4.00]	4374.00	0.319	**0.029**	0.085
Anankastia	2.67 [1.67, 3.67]	2.33 [1.00, 3.33]	4587.00	0.224	0.128	0.274
Psychoticism	2.67 [1.67, 3.67]	2.33 [1.67, 3.33]	4760.50	0.205	0.236	0.391
**BRIAN**						
Sleep	15.00 [13.00, 17.00]	14.00 [11.00, 16.00]	3639.50	0.563	**0.001**	**0.005**
Activity	15.00 [13.00, 17.00]	13.00 [11.00, 15.00]	3095.00	0.648	**<0.001**	**<0.001**
Social	11.00 [9.00, 13.00]	10.00 [9.00, 12.00]	4361.50	0.342	**0.042**	0.109
Eating	12.00 [9.00, 14.00]	10.00 [7.00, 12.00]	3923.00	0.423	**0.004**	**0.019**
Total Score	53.00 [45.75, 59.00]	47.50 [37.75, 54.00]	2985.00	0.664	**<0.001**	**<0.001**

Notes: *p* and *p*_FDR_ values < 0.05 are shown in bold. Abbreviations: ASRS v1.1 = Adult ADHD Self-Report Scale version 1.1; BIS-11 = Barratt Impulsiveness Scale-11; BRIAN = Biological Rhythms Interview of Assessment in Neuropsychiatry; HCL-32 = Hypomania Checklist-32; IQR = interquartile range; LPFS-BF 2.0 = Level of Personality Functioning Scale–Brief Form 2.0; *p*_FDR_ = *p*-value adjusted using the Benjamini–Hochberg false discovery rate procedure; PID5BF+ = Personality Inventory for DSM-5 Brief Form Plus; RIPoSt-40 = Reactivity, Intensity, Polarity, and Stability Questionnaire–40-item version; SMD = standardized mean difference; TEMPS-M = Temperament Evaluation of Memphis, Pisa, Paris, and San Diego–Münster version.

**Table 5:** Repeated-measures mixed-effects models testing the association of rating scale, sex, and ED with ADHD severity.

	Model A		Model B	
Term	Estimate (95% CI)	*p*	Estimate (95% CI)	*p*
Scale	−0.418 (−0.668; −0.167)	**0.001**	−0.340 (−0.598; −0.082)	**0.010**
Sex	−0.567 (−0.867; −0.267)	**<0.001**	−0.152 (−0.430; 0.126)	0.284
Scale × Sex	0.714 (0.386; 1.042)	**<0.001**	0.501 (0.173; 0.829)	**0.003**
ED	–	–	0.556 (0.313; 0.799)	**<0.001**
Scale × ED	–	–	−0.204 (−0.491; 0.083)	0.163
Sex × ED	–	–	0.195 (−0.098; 0.487)	0.191
Scale × Sex × ED	–	–	−0.295 (−0.640; 0.050)	0.093

Notes: Estimates are fixed-effect coefficients expressed in standardized units with 95% confidence intervals. Female sex and ASRS v1.1 were the reference categories. ED as measured by RIPoSt-NED was entered as a standardized continuous predictor. Both models were fitted on the same complete-case sample (*N* = 171 participants; 342 observations). *p* values < 0.05 are shown in bold. Abbreviations: ADHD = attention-deficit/hyperactivity disorder; ASRS v1.1 = Adult ADHD Self-Report Scale version 1.1; CAARS-O:SV = Conners’ Adult ADHD Rating Scales–Observer: Screening Version; CI = confidence interval; ED = emotional dysregulation; RIPoSt-NED = Reactivity, Intensity, Polarity, and Stability Questionnaire–Negative Emotion Dysregulation score.

## Data Availability

The data presented in this study are not publicly available due to privacy and ethical restrictions concerning sensitive clinical information and cannot be shared outside the research team. The raw data supporting the conclusions of this article can be made available by the corresponding author on request.

## Supplementary Materials

The following supporting information can be downloaded at: https://www.mdpi.com/article/10.3390/jcm15155954/s1, Missing-data analyses; Table S1: Sociodemographic, clinical, and first-degree family history characteristics in the overall sample, with missing-data proportions; Table S2: Lifetime psychiatric comorbidities in the overall sample, with missing-data proportions; Table S3: Observer- and clinician-rated measures of ADHD symptom severity, executive dysfunction, and psychosocial functioning in the overall sample, with missing-data proportions; Table S4: Self-reported measures of ADHD symptom severity, impulsivity, emotional dysregulation, affective temperament, personality functioning and traits, and biological rhythm disturbances in the overall sample, with missing-data proportions; Table S5: Linear regression models predicting ADHD severity after adjustment for treatment status or NED; Table S6: Mediation analysis: full path coefficients for the model Female sex → RIPoSt-NED → ASRS.

## Abbreviations

The following abbreviations are used in this manuscript:

ACME	Average Causal Mediation Effect
ADHD	Attention-Deficit/Hyperactivity Disorder
ASRS v1.1	Adult ADHD Self-Report Scale version 1.1
BIS-11	Barratt Impulsiveness Scale-11
BRI	Behavioral Regulation Index
BRIAN	Biological Rhythms Interview of Assessment in Neuropsychiatry
BRIEF-A	Behavior Rating Inventory of Executive Function–Adult Version
CAARS-O:SV	Conners’ Adult ADHD Rating Scales–Observer: Screening Version
CI	Confidence Interval
DIVA	Diagnostic Interview for ADHD in Adults
DIVA-5	Diagnostic Interview for ADHD in Adults, Fifth Edition
DSM-IV	Diagnostic and Statistical Manual of Mental Disorders, Fourth Edition
DSM-5-TR	Diagnostic and Statistical Manual of Mental Disorders, Fifth Edition, Text Revision
ED	Emotional Dysregulation
FAST	Functioning Assessment Short Test
FDR	False Discovery Rate
GEC	Global Executive Composite
HCL-32	Hypomania Checklist-32
ICD-11	International Classification of Diseases, Eleventh Revision
IQR	Interquartile Range
LPFS-BF 2.0	Level of Personality Functioning Scale–Brief Form 2.0
MI	Metacognition Index
NED	Negative Emotion Dysregulation
OLS	Ordinary Least Squares
PID5BF+	Personality Inventory for DSM-5 Brief Form Plus
REML	Restricted Maximum Likelihood
RIPoSt-40	Reactivity, Intensity, Polarity, and Stability Questionnaire, 40-item version
SMD	Standardized Mean Difference
TEMPS-M	Temperament Evaluation of Memphis, Pisa, Paris, and San Diego–Münster version
