# Supplementary material for: Sex Differences in Adult ADHD: A Multidimensional Multi-Informant Observational Study
Source: J Clin Med. 2026 Jul 30;15(15):5954. doi: 10.3390/jcm15155954 (PMC13467028; doi:10.3390/jcm15155954)
Supplement: Supplementary file 1 [file jcm-15-05954-s001.zip › jcm-4442354-supplementary.pdf]

Journal of Clinical Medicine

Missing-data analyses

Methods. For scores with more than 15% missing data, participants with and without available scores were compared on sex, age, ASRS v1.1 total score, and RIPoSt-40 NED. Age, ASRS v1.1 total score, and RIPoSt-40 NED were compared using Wilcoxon rank-sum tests, whereas sex distributions were compared using Pearson's chi-squared or Fisher's exact tests, as appropriate.

Results. Missing CAARS-O:SV and BRIEF-A scores were not associated with sex, age, ASRS v1.1 total score, or RIPoSt-40 NED (all $p > 0.20$). Missing FAST Occupational Functioning and total scores were associated with younger age (median 22 vs. 25 years and 22 vs. 26 years, respectively; both $p < 0.001$). Missingness in neither measure was associated with sex ($p = 0.867$ and $p = 0.911$, respectively), ASRS v1.1 total score ($p = 0.277$ and $p = 0.232$, respectively), or RIPoSt-40 NED ($p = 0.993$ and $p = 0.932$, respectively). Accordingly, analyses of these FAST measures were conducted on smaller available-case samples that were, on average, older than the overall sample.

Table S1. Sociodemographic, clinical, and first-degree family history characteristics in the overall sample, with missing-data proportions.

Variables	Missing (%)	Total (N = 212) Median [IQR] / N (%)
**Sociodemographic characteristics**		
Age (years)	0.0	24.00 [20.00, 29.00]
Education (years)	0.9	13.00 [13.00, 14.00]
Repeated grades	1.4	0.00 [0.00, 1.00]
Marital status	0.0	
Single		193 (91.0)
Married		15 (7.1)
Divorced		4 (1.9)
**Clinical characteristics**		
**Age at first referral (years)**	4.2	17.00 [10.00, 21.50]
**ADHD Presentation**	0.5	
Combined		103 (48.8)
Predominantly inattentive		103 (48.8)
Predominantly hyperactive/impulsive		5 (2.4)
**Treatment status (on treatment)**	9.0	39 (20.2)
**Family history (first degree)**		
Mood disorders	0.0	117 (55.2)
Anxiety disorders	0.0	54 (25.5)
Neurodevelopmental disorders	0.0	39 (18.4)
Substance use disorders	0.0	24 (11.3)
Suicide attempt	0.0	12 (5.7)
Psychotic disorders	0.0	6 (2.8)

Abbreviations: ADHD = attention-deficit/hyperactivity disorder; IQR = Interquartile Range

Table S2. Lifetime psychiatric comorbidities in the overall sample, with missing-data proportions.

Variables	Missing (%)	Total (N = 212)<br>Median [IQR] / N (%)
**Any mood disorder**	0.0	151 (71.2)
Age at onset (years)	34.4	16.00 [14.00, 20.00]
**Mood disorder**	0.0	
None		61 (28.8)
Bipolar I disorder		5 (2.4)
Bipolar II disorder		48 (22.6)
Cyclothymic disorder		84 (39.6)
Major depressive disorder		14 (6.6)
**Any anxiety disorder**	0.0	112 (52.8)
Separation anxiety disorder	0.0	21 (9.9)
Panic disorder	0.0	56 (26.4)
Agoraphobia	0.0	11 (5.2)
Social anxiety disorder	0.0	44 (20.8)
Generalized anxiety disorder	0.0	41 (19.3)
**Any obsessive-compulsive or related disorder**	0.0	16 (7.5)
Obsessive-compulsive disorder	0.0	7 (3.3)
Body dysmorphic disorder	0.0	10 (4.7)
**Any eating disorder**	0.0	32 (15.1)
Anorexia nervosa	0.0	6 (2.8)
Bulimia nervosa	0.0	6 (2.8)
Binge eating disorder	0.0	25 (11.8)
**Any substance use disorder**	0.0	58 (27.4)
Alcohol use disorder	0.0	25 (11.8)
Cannabis use disorder	0.0	45 (21.2)
Cocaine use disorder	0.0	21 (9.9)
Opioid use disorder	0.0	4 (1.9)
Sedative, hypnotic, or anxiolytic use disorder	0.0	5 (2.4)
Stimulant use disorder	0.0	9 (4.2)
**Any impulse-control/conduct disorder**	0.0	19 (9.0)
Oppositional defiant disorder	0.0	19 (9.0)
Conduct disorder	0.0	4 (1.9)
**Somatic symptom disorder**	0.0	8 (3.8)

Abbreviations: IQR = Interquartile Range.

Table S3. Observer- and clinician-rated measures of ADHD symptom severity, executive dysfunction, and psychosocial functioning in the overall sample, with missing-data proportions.

Variables	Missing (%)	Total (N = 212) Median [IQR]
**CAARS-O:SV**		
Inattention	17.9	16.00 [13.00, 21.00]
Hyperactivity/Impulsivity	17.9	10.00 [6.00, 15.00]
ADHD symptoms total	17.9	26.00 [19.00, 35.00]
ADHD Index	17.9	21.00 [16.00, 26.00]
**BRIEF-A (Informant)**		
Inhibit	18.4	15.00 [12.00, 17.00]
Shift	18.4	13.00 [11.00, 14.00]
Emotional Control	18.4	19.00 [16.00, 24.00]
Self-Monitor	18.4	11.00 [8.00, 13.00]
Initiate	18.4	18.00 [15.00, 20.00]
Working Memory	18.4	17.00 [14.00, 19.00]
Plan/Organize	18.4	21.00 [19.00, 24.00]
Task Monitor	18.4	13.00 [12.00, 15.00]
Organization of Materials	18.4	19.00 [16.00, 22.00]
Behavioral Regulation Index (BRI)	18.4	57.00 [50.00, 66.00]
Metacognition Index (MI)	18.4	89.00 [78.00, 98.00]
Global Executive Composite (GEC)	18.4	145.00 [130.00, 163.00]
**FAST (Clinician)**		
Autonomy	17.9	5.00 [3.25, 7.00]
Occupational Functioning	39.2	7.00 [5.00, 10.00]
Cognitive Functioning	16.0	8.00 [5.00, 10.00]
Financial Issues	16.5	3.00 [1.00, 4.00]
Interpersonal Relationships	17.0	8.00 [4.00, 11.00]
Leisure Time	16.0	2.00 [1.00, 3.75]
Total Score	41.5	33.00 [26.00, 43.00]

Abbreviations: ADHD = attention-deficit/hyperactivity disorder; BRI = Behavioral Regulation Index; BRIEF-A = Behavior Rating Inventory of Executive Function–Adult Version; CAARS-O:SV = Conners' Adult ADHD Rating Scales–Observer: Screening Version; FAST = Functioning Assessment Short Test; GEC = Global Executive Composite

Table S4. Self-reported measures of ADHD symptom severity, impulsivity, emotional dysregulation, affective temperament, personality functioning and traits, and biological rhythm disturbances in the overall sample, with missing-data proportions.

Variables	Missing (%)	Total (N = 212) Median [IQR]
**ASRS v1.1 Total**	0.0	46.00 [37.00, 54.00]
**BIS-11**		
Attentional	1.9	21.00 [19.00, 23.00]
Motor	2.4	26.00 [23.00, 28.00]
Non-Planning	3.8	28.00 [26.00, 30.00]
Total Score	3.8	74.00 [71.00, 78.00]
**RIPoSt-40**		
Affective Instability	1.4	47.00 [35.00, 58.00]
Emotional Impulsivity	0.5	29.00 [21.00, 36.00]
Negative Emotionality	0.5	42.00 [35.00, 49.00]
Positive Emotionality	0.5	40.00 [31.00, 47.00]
Negative Emotion Dysregulation	1.4	119.00 [93.00, 142.00]
**HCL-32**		
Factor 1 (Active/Elated)	1.4	12.00 [9.00, 15.00]
Factor 2 (Irritable/Risk-Taking)	2.4	5.00 [4.00, 7.00]
Total Score	2.4	17.00 [13.00, 22.00]
**TEMPS-M**		
Depressive	0.9	23.00 [19.00, 27.75]
Cyclothymic	0.9	24.00 [18.00, 30.00]
Hyperthymic	0.9	20.00 [16.00, 24.00]
Irritable	0.9	18.00 [12.00, 23.00]
Anxious	0.9	19.00 [13.00, 25.00]
**LPFS-BF 2.0**		
Self-Functioning	0.9	16.00 [13.00, 19.00]
Interpersonal Functioning	1.4	14.00 [11.00, 17.00]
**PID5BF+**		
Negative Affectivity	2.4	3.00 [2.33, 4.00]
Detachment	1.4	2.00 [1.33, 3.00]
Antagonism	3.8	1.67 [0.67, 2.67]
Disinhibition	1.9	3.00 [2.33, 4.00]
Anankastia	2.8	2.33 [1.33, 3.67]
Psychoticism	2.4	2.67 [1.67, 3.67]
**BRIAN**		
Sleep	4.7	15.00 [12.00, 16.75]
Activity	7.5	14.00 [12.00, 16.00]
Social	2.8	10.50 [9.00, 13.00]
Eating	3.8	10.50 [8.00, 13.00]
Total Score	9.4	50.00 [42.75, 56.00]

Abbreviations: ASRS v1.1 = Adult ADHD Self-Report Scale version 1.1; BIS-11 = Barratt Impulsiveness Scale-11; BRIAN = Biological Rhythms Interview of Assessment in Neuropsychiatry; HCL-32 = Hypomania Checklist-32; LPFS-BF 2.0 = Level of Personality Functioning Scale–Brief Form 2.0; PID5BF+ = Personality Inventory for DSM-5 Brief Form Plus; RIPoSt-40 = Reactivity, Intensity, Polarity, and Stability Questionnaire–40-item version; TEMPS-M = Temperament Evaluation of Memphis, Pisa, Paris, and San Diego–Münster version.

Table S5. Linear regression models predicting ADHD severity after adjustment for treatment status or NED.

**Models adjusted for treatment status**	**CAARS-O:SV ADHD symptoms total**		**ASRS v1.1 total score**	
**Variables**	**Estimate (95% CI)**	**p**	**Estimate (95% CI)**	**p**
Intercept	26.19 (23.76; 28.61)	**<0.001**	50.01 (47.45; 52.57)	**<0.001**
Sex (male)	1.46 (-1.71; 4.64)	0.364	-5.20 (-8.55; -1.85)	**0.003**
Treatment status (on treatment)	-0.06 (-4.11; 3.98)	0.975	-7.89 (-12.02; -3.76)	**<0.001**
**Models adjusted for NED**	**CAARS-O:SV ADHD symptoms total**		**ASRS v1.1 total score**	
**Variables**	**Estimate (95% CI)**	**p**	**Estimate (95% CI)**	**p**
Intercept	13.81 (6.65; 20.97)	**<0.001**	13.45 (8.01; 18.89)	**<0.001**
Sex (male)	3.44 (0.19; 6.68)	**0.038**	-1.34 (-3.88; 1.20)	0.300
RIPoSt-40 NED	0.09 (0.04; 0.15)	**<0.001**	0.28 (0.24; 0.32)	**<0.001**

Notes: Estimates are unstandardized regression coefficients with 95% confidence intervals. Models were fitted on model-specific complete cases: CAARS-O:SV model adjusted for treatment status, N = 174; ASRS v1.1 model adjusted for treatment status, N = 193; CAARS-O:SV model adjusted for RIPoSt-40 NED, N = 171; ASRS v1.1 model adjusted for RIPoSt-40 NED, N = 209. Female sex and no current treatment were the reference categories. RIPoSt-40 NED was entered as a continuous predictor. Abbreviations: ADHD = attention-deficit/hyperactivity disorder; ASRS v1.1 = Adult ADHD Self-Report Scale version 1.1; CAARS-O:SV = Conners' Adult ADHD Rating Scales–Observer: Screening Version; CI = confidence interval; NED = Negative Emotion Dysregulation; RIPoSt-40 = Reactivity, Intensity, Polarity, and Stability Questionnaire–40-item version.

Table S6. Mediation analysis: full path coefficients for the model Female sex → RIPoSt-NED → ASRS.

	Main analysis (N = 209)		Sensitivity analysis (N = 189)	
**Effect**	**Estimate (95% CI)**	**p**	**Estimate (95% CI)**	**p**
a path (Female sex → RIPoSt-NED)	18.76 (10.46; 27.06)	**<0.001**	12.90 (4.45; 21.35)	**0.003**
b path (RIPoSt-NED → ASRS)	0.28 (0.24; 0.32)	**<0.001**	0.26 (0.22; 0.31)	**<0.001**
c — total effect (Female sex → ASRS)	6.51 (3.18; 9.84)	**<0.001**	4.29 (0.75; 7.84)	**0.018**
c′ — direct effect (Female sex → ASRS ∣ RIPoSt-NED)	1.34 (-1.20; 3.88)	0.300	0.89 (-1.94; 3.73)	0.534
ACME — indirect effect (a × b)	5.17 (2.80; 7.57)	**<0.001**	3.40 (1.20; 5.74)	**0.001**
Proportion mediated	79.4%	–	79.2%	–

Notes: Estimates are unstandardized coefficients with 95% confidence intervals. The sensitivity analysis additionally adjusted for age, ADHD presentation, treatment status, and any lifetime mood disorder. Abbreviations: ACME = average causal mediation effect; ADHD = attention-deficit/hyperactivity disorder; ASRS = Adult ADHD Self-Report Scale v1.1; CI = confidence interval; RIPoSt-NED = Negative Emotion Dysregulation score of the Reactivity, Intensity, Polarity, and Stability Questionnaire–40-item version.
